# Fetal Tele-Echocardiography—An Approach to Improving Diagnosis and Management

**DOI:** 10.3390/diagnostics14222545

**Published:** 2024-11-13

**Authors:** Badreldeen Ahmed, Amal Elsisi, Justin C. Konje

**Affiliations:** 1 Feto Maternal Centre, Al Markhiya Doha, Doha P.O. Box 34181, Qatar; probadreldeen@hotmail.com; 2 Obstetrics and Gynaecology, Qatar University, Doha P.O. Box 2713, Qatar; 3 Obstetrics and Gynecology, Weill Cornell Medicine, Doha P.O. Box 24144, Qatar; 4 Paediatric Cardiology, Cairo University, Cairo 12613, Egypt; amal.elsisi@kasralainy.edu.eg; 5 Obstetrics and Gynaecology, Department of Health Studies, University of Leicester, Leicester LE1 7RH, UK

**Keywords:** tele-medicine, fetal echo, congenital heart diseases, zoom platform, prenatal diagnosis

## Abstract

***Introduction***: Antenatal diagnosis of cardiac abnormalities and counselling parents about postnatal care require a multidisciplinary team, which includes a paediatric cardiologist, a neonatologist, and a fetal medicine physician. Some of these kinds of expertise are not available in all centres with fetal medicine expertise. However, with modern technology, this could be provided remotely. Our objective was to assess the feasibility and outcomes of prenatal multidisciplinary tele-echocardiography diagnostic and counselling services. ***Materials and Methods***: Two centres based in separate countries provided a joint diagnostic and counselling service over a period of 14 months. The primary centre performed the fetal echocardiography with a Voluson E10 machine, and images were transmitted live using Zoom OPS system with video-consultation and counselling. The fetal echo was performed using the ISUOG Guidelines check list. ***Results***: There was an initial feasibility period of 2 months during which 10 women whose fetuses had normal hearts were scanned to test the workability of the system. Over a period of 12 months, 513 high-risk fetuses were then scanned, and out of these, 27 had congenital malformations. The most common were hypoplastic left heart syndrome (HHLS) and atrio-ventricular septal defect. Tele-echocardiography and counselling were successful in all the cases. Satisfaction with the service was 3.8/4, with the main limitation being the need for further referral to a tertiary centre for delivery. ***Conclusions***: Tele-echocardiography is reliable, and when combined with live counselling and support from a paediatric cardiologist, it is an option acceptable to patients. The greatest benefit was from being counselled by a team of experts at a single consultation rather than having to travel to another centre for consultation. With rapidly evolving technology, making video transmission easier and less expensive, we feel that consideration should be given not only to the development of tele-echocardiography but also to extending it to other aspects of fetal medicine.

## 1. Introduction

Abnormalities of the heart are the most common congenital anomalies and the leading cause of perinatal mortality from congenital anomalies [1,2]. A study in Western Europe found that about 45% of infant deaths from congenital anomalies were secondary to congenital heart defects (CHD) [3]. Similarly, these contributed to 35%, 37%, 42%, and 48% of deaths in Latin America, North America, Eastern Europe, and the South Pacific regions, respectively [3]. The prevalence of CHDs varies world-wide. In a population-based study in Northern England from 1998 to 2003, it was reported to be about 85.9/10,000 births and terminations, and 79.9/10,000 live births [4]. In Europe, data from EUROCAT (European Surveillance of Congenital Anomalies), the network of population-based registers of congenital anomalies in Europe, with a common protocol and data quality review, covering 1.5 million annual births in 22 countries, showed that CHDs were the most common non-chromosomal subgroup, at 65 per 10,000 births for the period of 2003–2007 [5]. In USA, CHDs are the most common congenital anomaly occurring in about 100/10,000 life births and are responsible for 4.2% of all neonatal deaths [6,7].

Despite the fact that CHDs contribute to the highest perinatal mortality from congenital malformations, prenatal detection rates vary widely in different geographic regions and for various types of CHDs, with fewer than one half of cardiac anomalies being identified before birth [8,9,10]. Some variations can be attributed to differences in operator ability, transducer frequency, patient body habitus, abdominal scars, gestational age, amniotic fluid volume, and fetal position [11,12,13,14,15]. In the UK, for example, the NHS Fetal Anomaly Screening Programme (FASP) sets a minimum standard detection rate of 50% for major cardiac abnormalities. Reported rates of prenatal diagnosis vary from 47 to 64% in the UK (47–59% for the period of 2015–2016 in Norfolk [16] and 52–50.4% for the period of 2021–2022 in the UK overall [17]) to 71.5% [18] in Southern France. In USA, prenatal detection rates have risen from 23% nearly 20 years ago [19] to approximately 35% [20].

It is generally accepted that prenatal diagnosis can improve birth outcomes, particularly for certain types of CHDs. It allows for prenatal awareness of the specific CHD by clinicians who can plan management postnatally, parental education to allow preparation for the birth of a neonate that will require specialized care and services, and the timing and place of delivery. Ideally, such antenatal management should be provided by a multidisciplinary team consisting of a fetal medicine physician, a paediatric cardiologist, a neonatologist, a clinical geneticist, and others (such as a cardiothoracic surgeon) where appropriate. There is robust evidence that such a set-up is associated with the best outcomes for fetuses diagnosed with CHDs in utero [21]. Such set-ups are limited to centres of excellence or university hospitals. However, a composite prenatal service is possible with the application of technology that is easily available. It is within this context that tele-ultrasonography has been shown to be extremely beneficial. Currently, this approach is being used to screen for congenital malformations—some with real-time imaging and others with a transmission of recorded images/videos for review. In the context of CHD, there are limited studies where this technology has been used beyond that of diagnosis. In a study in USA, there was evidence within New York that tele-echocardiography was not only useful in improving identification of CHDs but also in the provision of information to some of the women [22]. We run a tertiary-level diagnostic service in a private clinic where access to other kinds of expertise is unavailable on site. This means following diagnosis or suspicion of a congenital cardiac abnormality, the woman will have to be referred to a centre where multidisciplinary counselling can be provided. To overcome this, we set up a tele-echocardiography service with a paediatric cardiologist in a centre of excellence. Here, we report on the feasibility of this set-up, our experience over a period of 12 months, and the feedback from the women using the service.

## 2. Subjects and Methods

Our centre is a tertiary-level private fetal medicine centre manned by two sub-specialty trained fetal medicine physicians. There are two other obstetricians in the centre who refer patients to the fetal medicine specialists. The women attending the centre are either referred from other centres for a second opinion or attend primarily to receive most of their antenatal care. Some attend because of their history (diabetes, previous fetal abnormality or aneuploidy) for the specialist ultrasound scan evaluation of the fetus. The throughput in the unit varies from 4500 to 5200 cases per year.

### 2.1. Set-Up

The tele-echocardiography service was set up in two phases. In the first phase (a pilot), which ran for two months, a total of 10 women who agreed to tele-echocardiography were scanned in the set-up primarily to test feasibility. This was undertaken to ensure that transmission was of the highest quality. The women were scanned with a GE (Boston, MA, USA) Voluson E10 by one of two fetal medicine physicians [BA or JCK]). The other Tertiary Centre is based in Cairo where there is a Paediatric cardiologist (AE). Imaging is transmitted live to Cairo where the paediatric cardiologist (AE) sees the scanning, directs the acquisition of further planes (if necessary), and participates in the discussion and counselling with the on-site team and the patient. The standard approach to imaging the fetal heart (with a check list table) in our centre is that prescribed by ISUOG 2023 [15]. All the cases in this phase had normal hearts on imaging; hence, there was no need for the cardiologist to communicate in detail with the patients other than reassuring them that all was normal. Once we had refined the transmission (i.e., ensured that there were no connectivity or network problems during transmission) and were happy with the two-way reviews, discussions, and conclusions, we then embarked on the 2nd phase of the programme.

The second phase included women with fetuses diagnosed with a CHD as well as some with suspected but unconfirmed cardiac abnormalities. The data we present are for a period of 12 months from April 2022 to March 2023

### 2.2. Details of Transmission Set-Up

**Figure 1:**
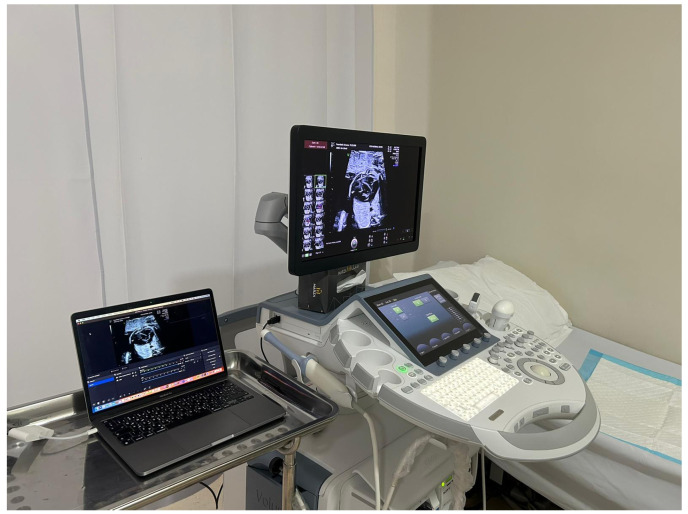
Set-up for imaging and tele-counselling. In this picture, the paediatric cardiologist has been replaced with an ultrasound image.

Figure 1 shows the set-up at the centre where the scanning was performed and then transmitted to Cairo (the recipient centre with the paediatric cardiologist). A free application (OBS Studio 30.1.2—https://obsproject.com) was downloaded onto a laptop, which was connected to the GE machine by and HDMI cable. The ZOOM (San Jose, CA, USA) platform was opened on the laptop and the screen shared to allow the paediatric consultant to link into the consultation and view the scanning images, which were also appearing on the laptop. As the application is free, and each session lasted no longer than 75 min, the ZOOM platform tended to be free as well.

### 2.3. Patient Satisfaction Survey

An anonymised patient satisfaction survey was undertaken for each of the women involved in the tele-echocardiography. The survey was based on a series of questions with answers rated on a 4-point Linkert scale (Table 1). For each of the variables (presented in the results) assessed, the woman was asked to score it based on the details in Table 1. Various questions were used for the survey. These questions centred on the information provided about the set-up, concerns about the involvement of an expert from a remote site in the imaging, and discussions, including addressing concerns and answering all questions, the quality of information provided, and the overall benefits of such a set-up. Each of the women was also asked about the option of being referred to the tertiary centre as opposed to having the tele-echocardiography service, as well as which aspect of the set-up they disliked most.

The cost savings were estimated by combining the approximate transport, consultation, and echocardiography costs. There was no additional cost for the tele-echocardiography service, although was this to be offered commercially, the consultation from the paediatric cardiologist would be factored into the cost.

## 3. Results

Over a period of 12 months, a total of 513 high-risk fetuses were scanned for congenital heart disease in our centre. A total of 4572 pregnant women were seen at our centre over this period. Of the 513 high-risk cases who underwent fetal echography, 27 were diagnosed with congenital cardiac defects for an incidence of 5.3/100 or 53/10,000. Table 2 shows the demographics of these women. Values are presented as mean and range as the data were normally distributed. There were no differences in the demographics of mothers whose fetuses had a cardiac abnormality and those whose fetuses did not.

Table 3 shows the spectrum of abnormalities diagnosed. The most common were hypoplastic heart (8) and atrioventricular septal defect (7). There were two cases in which the diagnosis was modified by tele-echocardiography. These were initially classified as complex cardiac abnormalities but were later refined after specialist consultation.

Good-quality images were obtained and transmitted to the cardiologist in all the cases. Counselling was jointly provided by the obstetrician and the paediatric cardiologist. The average time for each patient was 47 min (range 30–65 min). There were no technical issues encountered with the transmission of the images and counselling of the couple.

Table 4 shows the survey results for the women. All the 27 women with suspected/diagnosed fetal cardiac abnormality completed the questionnaire. The overall score for this service was 3.8 out of 4. The aspect of the set-up that was least liked was the fact the women had to be referred to the tertiary hospital despite these multidisciplinary sessions. They would have preferred contacts with the team at the tertiary hospital at this set-up. Their anxiety was primarily because of fear of meeting new people and the possibility of being offered conflicting information and counselling. The quality and quantity of information provided was judged to be appropriate by everyone; they were all happy with the communication at these sessions. The time spent during the session was considered just adequate. The women, overall, felt very comfortable asking questions to the cardiologist although this was virtual. To most of them, it was like having a video phone call with friends or family.

The estimated average cost savings for each woman that covered transport, consultation, and echocardiography was USD 749. Time off work to attend an additional assessment was not included in this estimate. The paediatric consultation cost was approximately USD 200.

## 4. Discussion

Telehealth is defined as health actions carried out remotely, including consultation, diagnosis, therapeutic guidance, monitoring, and referral of patients [23,24]. It has been shown to be feasible and highly effective in fetal ultrasound examination and mostly in relation to linking rural areas with experts in centres of excellence [25,26,27,28,29,30,31,32]. Our set-up provides a virtual setting for multidisciplinary antenatal care for a highly specialised service. An important consideration for such a set-up is time difference. In our setting, this was not an issue as there are no time differences between the centres, but where there is, adjusting for this may incur additional costs, which must be factored into the modelling and delivery.

In the context of fetal echocardiography, telemedicine offers an alternative referral strategy, whereby digitised images are transmitted electronically [33] with the potential to decrease time to diagnosis. In some cases, there is teleconsultation in real time (where the sonographer scans and the remote specialist views the live images) [32,34,35] or via transmission of pre-recorded ultrasound images (store-and-forwarded) in the absence of the woman [36]. Stored-and-forwarded telemedicine has also been shown to be a reliable and acceptable first consultation for families with well-recognised risk factors for CHD and for women screened with a suspected fetal heart anomaly [36]. Dowie et al. found no false-negative cardiac diagnoses and no significant differences in the antenatal mean costs until delivery for the cohort assessed by telemedicine compared with those referred for direct assessment [36].

The quality of the live transmissions in this set-up was just as good as face-to-face scanning. An additional benefit for the fetal medicine physicians was the help provided by the paediatric cardiologist in redefining the abnormalities through varied scanning planes and discussing the rationale for altering or refining the diagnosis. This was extremely beneficial to the fetal medicine physicians—it allowed for improvement in echocardiography techniques and, furthermore, enabled appreciation of specific counselling on not only the diagnosis but the course of the abnormalities postpartum as well as their management. In fact, Fernandes et al. [37] concluded that a successful telemedicine program can also be beneficial to the professionals, the patients, and the health system itself.

In most of the cases, the diagnosis was accurately made by the fetal medicine specialist. The paediatric cardiologist created a plan for immediate postnatal care. The type of intervention was discussed, including interventional cardiac catheterisation or open-heart surgery. We show and confirm that a complex prenatal service such as imaging and counselling for cardiac abnormalities by tele-echo is feasible. Such a set-up could be replicated in other areas that are not fortunate enough to be able to provide combined on-site services. All patients were satisfied with the care received and valued the one-step approach for diagnosis and discussion of the plan of action after delivery.

Most previous fetal tele-echocardiography services concentrated on improving the diagnosis of congenital cardiac abnormalities (which has traditionally been low). In such set-ups, images and/or videos are obtained by trained sonographers/obstetricians and transmitted to cardiologists/fetal medicine obstetricians for review. While these have consistently shown significantly higher detection rates for CHD, the women would invariably have to travel to centres of excellence for counselling. In a cost-effective analysis, Mistry and Gardiner [38] showed that providing tele-echo services were cost effective not only in high-risk cases but as a part of the antenatal anomaly service. In that particular set-up, the involvement of a paediatric cardiologist made the counselling more effective.

Our set-up combines the confirmation of abnormalities, refining of diagnosis, and counselling. The benefits of this approach are enormous, as shown by the satisfactory scores from the patients. Interestingly, none of the women who were managed in this set-up had problems with the virtual counselling.

In a study in Kentucky, involving small regional hospitals and a paediatric referral centre, a fetal tele-echo service was shown to be statistically associated with a significant increase in the rate of prenatal diagnosis of CHD. This study concluded that fetal tele-echocardiography is an effective method that improves the rate of prenatal diagnosis in regions served by small regional hospitals with limited access to fetal echocardiography [22].

The technology used for this set-up is simple and should be possible in most units where there is WIFI, with the adoption of teleconferencing platforms such as ZOOM. However, concerns must remain about ensuring confidentiality of such virtual consultation. The accuracy of this set up was in keeping with reported rates. Brown et al. [39], for example, reported a specificity and a sensitivity of 100% for complex congenital heart disease, and a sensitivity of 100% and a specificity of 79% for the screening of less serious congenital heart diseases.

An important consideration in the provision of such a service is cost. For the women, there was a significant cost saving. Additional indirect costs not included were those of time off work for the additional service. In countries where there are costs associated with paediatric cardiologists working in tertiary paediatric cardiology units (or fetal medicine centres), some patients may struggle to meet the cost of such a service, especially where there is no insurance cover. This could be compounded by travel costs—a factor that was not very crucial in our own cohort. Additional to this would be the wait to see the specialist, which is associated with significant parental anxiety.

## 5. Limitations

While this set-up was highly valued and rated by the patients and the team involved in their care, there were several limitations. Firstly, these women still had to be referred to a tertiary centre for their intrapartum and postnatal care. This meant undergoing further imaging to confirm our findings and followed again by counselling. Although this may seem a duplication, we feel that there are advantages to this—firstly the diagnosis had been confirmed by a paediatric cardiologist, and it was, therefore, unlikely that this would be changed (and indeed this was unchanged in all the cases); and secondly, the parents would have already been prepared for the counselling and management, as this had already been provided in out set-up. A third limitation, though minor, is the fact that our patients, going to the referral centre, would have had to see a completely new team with its own problems. An ideal setting would be one involving the tertiary centre at the time of diagnosis where the delivery and postnatal care will be provided. Integrating the intrapartum/postnatal care centres in set-ups will address this limitation.

Although we were informed by the women of the outcome of the deliveries, we did not have a formal follow-up of these pregnancies. Including this in our setting would allow for a much robust evaluation of the service. This should be taken into consideration when planning this service.

Another limitation was the fact that some of the patients had to make a second trip to our centre for this service following the diagnosis. An ideal set-up would be one where a tele-echo multidisciplinary service is offered once the diagnosis is made or suspected. Logistically, this was difficult to arrange but in the long term, we plan to run this clinic in tandem with the paediatric cardiologist so that all consultants will be contemporaneous.

Finally, we acknowledge that the numbers are small but hope that having demonstrated the feasibility and benefit of tele-echocardiography, more data would be generated to validate our findings. Ours was primarily aimed at assessing feasibility and subsequently should provide a more thorough evaluation of the economic implications/benefits of such a service.

## 6. Conclusions

We have been able to demonstrate the feasibility of a multidisciplinary tele-echocardiography diagnostic and counselling service with minimal need for additional resources. The benefits include a more refined diagnosis, education for physicians at the primary site, and benefits to the women in terms of counselling and preparing them for postnatal care (i.e., giving information not only about the abnormality but also about the postnatal treatment and course of the abnormality, including its impact on quality of life) information to be provided at the centres where delivery will occur and allay any anxieties about diagnosis. Patient satisfaction was excellent; however, limitations included the need for a second visit for this consultation and the knowledge that further counselling by a new team would be provided at the centre of excellence where delivery and postnatal care would take place. We feel that this approach could be extrapolated to rare malformations with teams based on different continents, such as congenital diaphragmatic hernia and spina bifida where in-utero surgery is an option.

## Figures and Tables

**Table 1:** A 4-point Linkert scale.

Point	Scale Range	Interpretation (with Regards to Experience)
4	4.00–3.00	Excellent service (no negatives on the service)
3	2.99–2.00	Good service (one negative on the service)
2	1.99–1.00	Average service (2–3 negatives on the service)
1	1.00–0.99	Poor service (>3 negatives on the service)

**Table 2:** Patient demography.

Variable	Congenital Cardiac * Abnormality (*N* = 27)	Normal Heart (*N* = 486) *
Age (years)	33 (26–43)	29 (23–44)
BMI	26.8 (19.5–45.8)	25.8 (19.6–50.6)
Gravidity	4 (1–11)	3 (1–12)
GA at imaging	21 (19–24)	19 (18–22)

GA = gestational age. BMI = body mass index. * *p* > 0.05 for all the variables when the two groups are compared.

**Table 3:** Spectrum of congenital heart abnormalities over the period.

Congenital Malformation	No (%)
Hypoplastic left heart syndrome	8 (29.6)
* Atrioventricular septal defect	7 (25.9)
Ventricular septal defects	4 (14.8)
Tricuspid atresia	3 (11.1)
Transposition of the great arteries (TGA)	3 (11.1)
Complex cardiac abnormalities	2 (7.4)

* Some of the cases were suspected but confirmed by the paediatric cardiologist.

**Table 4:** Mean score from patient survey (scale of 1–4).

Variable	Score Obtained Out of 4
Comfort with the set up	3.6
Communication of findings	3.8
Amount of information received at counselling	3.9
Understanding video counselling	3.5
Time to teleconsultation	3.4
Willingness of cardiologist to answer questions	3.8
Comfortable talking to the cardiologist virtually	3.7
Overall rating of the set-up and service	3.8
Would you have preferred referring to tertiary centre for the consultation	1.2
Aspects that disliked most Waiting for the appointment Knowing that I will have to go and meet another team (in the hospital) to look after the baby	2.8 1.3

## Data Availability

The original contributions presented in the study are included in the article, further inquiries can be directed to the corresponding author.
